# Essentials of Homœopathic Materia Medica

**Published:** 1894-04

**Authors:** 


ESSENTIALS OF HOMŒOPATHIC MATERIA MEDICA; being a Quiz Compend upon the Principles of Homœopathy, Homœopathic Pharmacy, and Homœopathic Materia Medica, arranged and compiled especially for the use of students of medicine, by W. A. Dewey, M. D., late Professor of Materia Medica in Hahnemann Hospital, College of San Francisco, etc. Philadelphia: Bœricke & Tafel, 1894. Price, $1.50 net.

This little volume is, as stated in the preface, designed especially for students in homœopathic colleges as an aid to the study of homœopathic materia medica.

It is arranged in the form of question and answer, and the different remedies are divided into groups; those derived from the Vegetable Kingdom; those derived from the Animal Kingdom; those derived from the Mineral Kingdom, and the Nosodes. The first chapter gives the principles of Homœopathy, the best views on the value of symptoms, provings, aggravations, ameliorations, and the chronic miasms. These questions are all treated on a soundly homœopathic basis, and therefore the book is a safe one to put into the hands of the beginner. The second chapter treats of the Homœopathic Pharmacy, while the succeeding chapters are devoted to the individual remedies according to the groupings already given. Nothing but the essentials of the remedies is given. The author is the same who in conjunction with Dr. Bœricke edited the third edition of the Twelve Tissue Remedies of Schüssler which was reviewed by the present writer in this journal for May, 1893, page 300.

As Dr. Dewey had shown his comprehension of Homœopathy in the editing of *The Tissue Remedies*, it will readily be believed that in the work now on review he has contributed an acceptable addition to homœopathic literature.

